# Expression of mdr1 and gst-pi in human breast tumours: comparison to in vitro chemosensitivity.

**DOI:** 10.1038/bjc.1990.160

**Published:** 1990-05

**Authors:** W. N. Keith, S. Stallard, R. Brown

**Affiliations:** CRC Department of Medical Oncology, Glasgow, UK.

## Abstract

**Images:**


 © Macmillan Press Ltd., 1990

### Expression of *mdr1* and *gst-π* in human breast tumours: comparison to *in vitro* chemosensitivity

W.N. Keith, S. Stallard & R. Brown

*CRC Department of Medical Oncology, Garscube Estate, Switchback Road, Glasgow G61 1BD, UK.*

**Summary** Increased expression of the *mdr1* gene, encoding the 175 kDa P-glycoprotein, and the *gst-π* gene, encoding the anionic isozyme of glutathione S-transferase (GST), have previously been detected in continuous human breast cancer cell lines selected *in vitro* for resistance to doxorubicin. In this present study we have measured RNA levels of *mdr1* and *gst-π* in primary human breast tumour biopsies prior to chemotherapy and from tumours which have different inherent responses to doxorubicin treatment, including colon, head and neck squamous cell carcinomas and myeloid leukaemias. Detectable levels of *mdr1* mRNA was observed in 25 out of 49 breast tumours, with up to a 100-fold range in expression. A narrower range of *gst-π* expression has also been observed in these tumours. Chemosensitivity of cells grown in short-term culture from some of the breast tumours has been measured by an *in vitro* colony forming assay in the presence of doxorubicin. Comparison of the dose of doxorubicin causing 50% inhibition of growth ($ID_{50}$) with RNA levels showed that the tumours with high *mdr1* expression had high $ID_{50}$, while the more sensitive explants had low *mdr1* expression. These results support a role for *mdr1* gene expression in determining the response of human breast cancer cells to chemotherapy.

Doxorubicin is known to be active against a range of solid tumours and is one of the best available agents in the treatment of breast cancer (Bonadonna *et al.*, 1970; O'Bryan *et al.*, 1973; Blum & Carter, 1974). However, both inherent and acquired resistance are in many cases major obstacles in successful treatment (O'Bryan *et al.*, 1977). Increased levels of expression of the *mdr1* gene, which codes for a 175 kDa plasma membrane associated glycoprotein, and the *gst-π* gene, encoding the anionic isozyme of glutathione S-transferase (GSTπ) have previously been shown in doxorubicin (adriamycin) resistant cell lines, derived by drug selection in culture (Kartner *et al.*, 1985; Riordan *et al.*, 1985; Scotto *et al.*, 1986; Cowan *et al.*, 1986; Deffie *et al.*, 1988; Van der Bliek *et al.*, 1988). Many of these cell lines have been shown to be cross resistant to other chemotherapeutic agents such as Vinca alkaloids, anthracyclines and epipodophyllotoxins and have been termed multidrug resistant (MDR). The 175 kDa plasma membrane-associated protein, P-glycoprotein, has been suggested to increase resistance to a variety of drugs by increasing their efflux from the cell (Gerlach *et al.*, 1986; Chen *et al.*, 1986). GST isozymes have in common the ability to catalyse conjugation of drugs to reduced glutathione leading to drug detoxification (Jakoby, 1978).

A range of expression of *mdr* and *gst-π* has been shown in human tumour samples, both between tumours derived from different tissues and derived from the same tissue (Goldstein *et al.*, 1989; Gerlach *et al.*, 1987; Moscow *et al.*, 1989). However, the relationship between levels of expression of *mdr1* or *gst-π* and clinical drug resistance has yet to be fully elucidated. The identification of particular mechanisms of resistance in a tumour type is important for treatments designed to circumvent resistance (Kaye, 1988). In this present study we have measured RNA levels of *mdr1* and *gst-π* in untreated human breast tumour biopsies. We have compared the expression of these genes in breast cancer with other tumour types including colon tumours, head and neck squamous cell carcinomas and leukaemias, as well as cell lines selected for resistance to doxorubicin. We have also analysed epithelial cells grown during short term culture of breast tumour biopsies for *in vitro* sensitivity to doxorubicin, as measured by a colony forming assay (Smith *et al.*, 1981*a,b*). The response of cells to doxorubicin compared to *mdr1* and *gst-π* mRNA allows an indirect analysis of whether these mechanisms are involved in response of breast tumour cells to doxorubicin.

Correspondence: R. Brown.
Received 8 September 1989; and in revised form 3 January 1990.

### Materials and methods

### *RNA extraction from tumour biopsies*

Tumour biopsies were frozen in liquid nitrogen immediately after surgical removal and were stored frozen at − 70°C. All solid tumour samples were from patients who had not received any chemotherapy. Routine histology of the samples confirmed that the majority of the biopsy consisted of tumour cells. Solid tumours were pulverised whilst still frozen in a micro-dismembrator II (Braun, FRG) and DNA and RNA extracted by cell lysis and phenol/chloroform extraction (Kreig *et al.*, 1983). Nucleated cells were isolated from the leukaemic samples by ficoll gradient centrifugation and RNA/DNA isolated as described above. DNA was removed from the samples by digestion with RNase-free DNase I (BCL, molecular biology grade). The quantity and quality of the RNA was initially assessed by the presence of ribosomal 28S and 18S RNA after electrophoresis in agarose.

### *Hybridisation probes*

An *mdr1* probe, mdr5A, which encodes about one-third of the coding region of a full-length *mdr1* cDNA has previously been described (Ueda *et al.*, 1987*b*). One μg of BamH1 digested mdr5A was used as substrate for a riboprobe transcription kit (BCL) in the presence of uridine 5′ $\alpha$-$^{32}$P-triphosphate (30 TBq $mmol^{-1}$) from Amersham and SP6 polymerase. A full-length cDNA of *gst-π*, pGPi (Kano *et al.*, 1987), was cloned into the EcoRI site of the polylinker of the riboprobe vector Bluescribe (Pharmacia). One μg of HindIII digested pGPi probe was used as substrate for riboprobe transcription as described above. The 7S probe (Balmain *et al.*, 1982) was labelled with cytosine 5′ $\alpha$-$^{32}$P-triphosphate (111 TBq $mmol^{-1}$) from Amersham by nick translation and Poly d(T) probe was made by kinase labelling poly $d(T)_{18-20}$ oligonucleotide (Pharmacia) with adenosine 5′ $\gamma$-$^{32}$P-triphosphate (111 TBq $mmol^{-1}$) from Amersham.

### *Hybridisation analysis*

*Northern hybridisation* The RNA was electrophoresed in 1.4% agarose/6% formaldehyde gels and transferred to Gene Screen membranes (NEN/DuPont). Hybridisation was in 50% formamide buffer as previously described (Shen *et al.*, 1986) for 16 h at 57°C with $5 \times 10^6$ c.p.m. of synthetic RNA per ml. The filters were washed with 2 × SSC/0.1% SDS for 15 min at room temperature followed by two 20 min washes at 65°C with 0.1 × SSC/0.1% SDS. Autoradiograms were exposed for 1–3 days. Amounts of RNA on the filters were quantified by reprobing the same filter with a probe for 7S RNA and intensity of signal measured by densitometrical scanning.

*Dot blot hybridisation* Gene screen filters were presoaked in 0.25 M di-sodium phosphate buffer and 15, 5, 1.6 and 0.5 μg of total RNA applied. After baking, prehybridisation, hybridisation and washing were performed exactly as described above for the Northern hybridisation experiments. Amounts of RNA on the filters were quantified by reprobing the same filter with a kinase labelled poly d(T) oligomer as previously described (Harley, 1987). Repeat identical RNA from tumours, MDR cell lines and their parental drug sensitive cell lines which had a range of both *mdr1* and *gstπ* expression were analysed on all filters to allow comparison between experiments. Autoradiograms were exposed overnight and intensity of signals were quantified using densitometrical scanning.

*Colony forming assay for doxorubicin sensitivity of breast tumour outgrowths*

Sensitivity of breast cells to doxorubicin was assessed using the short-term colony forming assay described by Smith *et al.* (1981*b*, 1985). Tumour tissue was minced and disaggregated in medium containing 200 U $\mu l^{-1}$ collagenase (Worthington). Resultant ductal alveolar structures and clumps of cells were plated onto plastic and allowed to grow for 7–10 days. Growth was supported in nutrient medium F10/DMEM supplemented with $10^{-9}$ M oestradiol, 10 μg $ml^{-1}$ insulin, 5 μg $ml^{-1}$ transferin and 5 mg $ml^{-1}$ epidermal growth factor. Outgrowth cells were then trypsinised, counted and plated onto mitomycin C (4 mg $ml^{-1}$) treated STO mouse fibroblast feeder layers. They were then exposed to a range of eight concentrations of doxorubicin (from $5 \times 10^{-6}$ M to $3.4 \times 10^{-11}$ M) for 24 h. Drug was removed, the nutrient medium replaced and surviving colonies counted after 2 weeks. Sensitivity was determined by the amount of drug required to kill 50% of the control untreated cells ($ID_{50}$). Greater detail of this assay and analysis of a large number of breast tumours will be published separately (S. Stallard, in preparation).

### Results

### mdr1 *mRNA levels in human tumours*

RNA prepared from human tumours and cell lines were analysed by dot-blot hybridisation using the same conditions of stringency that detect specific 4.5 kb *mdr1* mRNA transcripts and 0.7 kb *gst-π* transcripts in Northern hybridisations. Typical dot-blots and Northern hybridisations using each probe are shown in Figure 1. Of 49 primary breast tumour biopsies from untreated patients, 25 samples had measurable levels of *mdr1* mRNA (Figure 2). High levels of *mdr1* mRNA were also detected in one breast tumour lymph node biopsy. The levels of *mdr1* mRNA in some of these tumours reached levels equivalent to that detected in cell lines selected for drug resistance *in vitro*. Signal intensity of 20 μg RNA from the cell lines MCF-7ADR (Cowan *et al.*, 1986), A2780AD (Rogan *et al.*, 1984) and H69LX10 (an adriamycin resistant cell line isolated from a human lung cell line; P. Twentyman, personal communication) had values of 90, 160 and 100 respectively. Dot blot hybridisations contained RNAs from the adriamycin resistant cell line, H69LX10 and the parental drug sensitive line, H69, as well as repeat samples of the tumour RNAs to allow comparison between hybridisation experiments as described in the Materials and methods. Undetectable and very low signal intensities were given an arbitrary value of 1.

Biopsy samples from untreated colon tumours all showed detectable *mdr1* mRNA. The range of *mdr1* levels in the colon tumours overlapped with about 20% of the breast tumour samples. Of fourteen untreated squamous cell carcinomas of the head and neck, eight had measurable levels of *mdr1* mRNA. Detectable *mdr1* mRNA was also present in three out of five untreated acute myeloid leukaemia (AML) samples. The chronic myeloid leukaemia (CML) samples shown represent sequential samples from the same patient. The sample with undetectable *mdr1* RNA was taken prior to chemotherapy, while the sample with detectable expression was taken after anthracycline based chemotherapy.

**Figure 1** Expression of *mdr1* and *gst-π* in cell lines and tumour biopsies. **a**, Northern blot filter of cell line RNAs hybridised with labelled mdr5A probe, showing specific *mdr1* 4.5 kb mRNA. Sizes were determined by running in parallel molecular mass markers from an RNA ladder (Bethesda Research Laboratories). Lanes 2, 4 and 6 are adriamycin resistant cell lines H69LX10, MCF7ADR and A2780AD; lanes 1, 3 and 5 are the corresponding parental cell lines. **b**, The filter shown in A was stripped of hybridising probe and rehybridised with labelled GPi probe, showing specific *gst-π* 0.7 kb mRNA. The filter was then reprobed with 7S RNS probe to show equivalent loading of samples (data not shown). **c**, Dot-blot hybridisation of tumour RNAs probed with mdr5A, GPi and poly d(T). Lane a is H69LX10, lane, b-l are breast tumour RNAs.

### gst-π *mRNA levels in human tumours*

Figure 3 shows the *gst-π* mRNA levels detected in the same tumour samples as shown in Figure 2. Signal intensity of 20 μg RNA from the cell lines MCF-7ADR (Cowan *et al.*, 1986), A2087AD (Rogan *et al.*, 1984) and H69LX10 (an adriamycin resistant cell line isolated from a human lung cell line; P. Twentyman, personal communication) had values of 60, 55 and 10 respectively. Dot blot hybridisations contained RNAs from the adriamycin resistant cell line, H69LX10 and the parental drug sensitive line, H69, as well as repeat samples of the tumour RNAs to allow comparison between hybridisation experiments as described in the Materials and methods. Undetectable and very low signal intensities were given an arbitrary value of 1. All the breast tumour samples, with the exception of one, showed low or undetectable levels of *gst-π* mRNA. On the other hand all of the colon tumours, with the exception of one, showed relatively high levels of transcripts. The leukaemia samples generally had low levels of *gst-π*, including the sequential CML samples taken during chemotherapy. A large proportion of squamous cell carcinomas of the head and neck had high levels of *gst-π* mRNA levels.

**Figure 2** Quantitation of *mdr1* expression in tumour samples. Results obtained by dot-blot hybridisation with the mdr5A probe are graphically displayed. Inverted triangles, untreated breast tumours (filled symbols are lymph node biopsies); diamonds, colon tumours; squares, squamous cell carcinomas of the head and neck; open triangles, acute myeloid leukaemia; filled triangles, chronic myeloid leukaemia. Undetectable and low levels of *mdr1* expression are given an arbitrary value of 1. Adriamycin resistant cell lines MCF7ADR, A2780AD and H69LX10 have values of 90, 160 and 100 respectively on this scale.

**Figure 3** Quantitation of *gst-π* expression in tumour samples. Results obtained by dot-blot hybridisation with the GPi probe are graphically displayed. Symbols are as in Figure 2. Undetectable and low levels of *gst-π* expression are given an arbitrary value of 1. Adriamycin resistant cell lines MCF7ADR, A2780AD and H69LX10 have values of 60, 55 and 10 respectively on this scale.

**Figure 4** *mdr1* and *gst-π* RNA levels and *in vitro* chemosensitivity of breast tumours explants. RNA levels are as described in Figures 2 and 3. The $ID_{50}$ of each sample is the concentration of doxorubicin giving 50% surviving colonies of epithelial cell outgrowths from breast tumour biopsies.

*Comparison of* mdr1 *and* gst-π *mRNA levels to* in vitro *sensitivity of cell outgrowths of breast tumours*

Comparison of doxorubicin $ID_{50}$ to *mdr1* and *gst-π* mRNA level is shown in Figure 4 for 14 breast tumour biopsies and two normal breast tissue biopsies. No sample which is relatively sensitive to doxorubicin was observed to have high *mdr1* or *gst-π* mRNA level. All of the samples with high *mdr1* levels have relatively high $ID_{50}$ for doxorubicin. The level of *mdr1* expression weakly correlates with $ID_{50}$ to doxorubicin with a Pearson correlation coefficient ($r$) of 0.46 ($P < 0.1$). These observations support a role of *mdr1* expression in response of epithelial cells derived from breast tumours to doxorubicin.

No significant correlation between $ID_{50}$ to doxorubicin and *gst-π* expression using the Pearson correlation test was observed. Each individual tumour sample has been numbered in Figure 4 to allow comparison between *mdr1* and *gst-π* expression. Sample number 13 showed high levels of *mdr1* and *gst-π* expression. Comparison between *mdr1* and *gst-π* mRNA levels in all the breast samples assayed shows a low level of correlation which is only slightly significant ($r = 0.48$, $P < 0.1$). Thus for some of these tumours coexpression of *mdr1* and *gst-π* may be occurring, as has been observed for adriamycin resistant MCF7 breast cell lines (Cowan *et al.*, 1986).

### Discussion

Doxorubicin is widely used in the treatment of advanced breast cancer, with an overall response rate among breat cancer patients of about 55% (Blum & Carter, 1974; O'Bryan *et al.*, 1977). This means that almost half the patients are resistant to doxorubicin from the outset of treatment. A better understanding of the mechanisms underlying this resistance should lead to improved therapeutic results. Drug delivery studies in breast cancer patients suggest that defective delivery of adriamycin into breast tumours is unlikely to be a major factor (Stallard *et al.*, 1990). Increased *mdr* and *gst-π* expression have been detected in continuous human breast cancer cell lines selected *in vitro* for resistance to doxorubicin (Cowan *et al.*, 1986; Moscow *et al.*, 1988). Several lines of evidence support the involvement of *mdr1* expression in resistance of tumours to chemotherapy: (a) full-length cDNAs for the *mdr1* gene transfected (Ueda *et al.*, 1987*a*) or infected (Guild *et al.*, 1988) into cells confer multidrug resistance; (b) tumour types which are clinically drug resistant, such as colon, generally have elevated *mdr1* mRNA levels (Goldstein *et al.*, 1989; Fojo *et al.*, 1987*b*); (c) cell lines from tumours with elevated *mdr1* mRNA levels have a multidrug resistant phenotype which is reversible by inhibitors of the multidrug transporter such as verapamil and quinidine (Fojo *et al.*, 1987*a*); (d) immunocytohistochemical staining for P-glycoprotein expression correlates with *in vitro* sensitivity to doxorubicin of tumour tissue from patients (Salmon *et al.*, 1989).

We have detected *mdrl* mRNA in 25 out of 49 primary breast tumour biopsies from untreated patients. Detectable *mdrl* expression has also been observed in one out of three lymph node biopsies. In these untreated tumours a hundred fold range in expression of *mdrl* has been observed with some samples reaching the levels observed in resistant cell lines selected *in vitro*. Levels of *mdrl* expression in about 20% of the breast tumour samples are equivalent to the levels we detect in intrinsically chemoresistant colon tumours. These results support a possible involvement of *mdrl* expression in response to chemotherapy of some breast tumours.

*In vitro* chemosensitivity to doxorubicin of epithelial cells grown from breast tumour biopsies showed that no sample which is relatively sensitive to doxorubicin was observed to have high *mdrl* or *gst-π* mRNA level. All of the samples with high *mdrl* levels have relatively high $ID_{50}$ for doxorubicin. These observations are supportive of a role for *mdrl* expression in limiting the response of breast cells to doxorubicin. A number of tumour samples however are relatively resistant to doxorubicin, yet have low levels of *mdrl* expression. In these tumours alternative resistance mechanisms may be effective. Increased expression of *gst-π*, co-expressed with *mdrl*, has been observed in some doxorubicin resistant breast tumour cells (Cowan *et al.*, 1986). With one exception, we have detected low levels of *gst-π* mRNA in the breast tumour samples. No large differences in expression levels were detected and no significant correlation with doxorubicin sensitivity of outgrowths was observed. However, it is still possible that *gst-π* expression may have a role in chemoresponsiveness of a subset of breast tumours. A weak correlation exists between expression of *mdrl* and *gst-π* in the breast tumour samples we have analysed, suggesting that common mechanisms may be involved in their expression.

Our results show that *mdrl* expression can be detected in approximately half of the breast tumours analysed and that explants from tumours with high *mdrl* expression are relatively more resistant to doxorubicin *in vitro*. These results are in agreement with those of Salmon *et al.* (1989), who recently showed that five out of 13 breast tumours stained positively with a P-glycoprotein antibody and that all five of these tumours were 'resistant' using a short-term *in vitro* assay of doxorubicin sensitivity. Goldstein *et al.* (1989) have found detectable levels of *mdrl* mRNA in nine of 57 untreated breast tumour biopsies. However, Merkel *et al.* (1989) found no *mdrl* mRNA in 53 untreated breast tumour samples. The differences in proportions of tumours with detectable *mdrl* mRNA may be due to technical variations in sensitivity of the hybridisation conditions used. The stringent hybridisation conditions used in this present study detect only the *mdrl* specific 4.5 kb mRNA and appropriate positive and negative control RNAs were included in hybridisation experiments. However, the differences in detection of *mdrl* mRNA may also represent variations in *mdrl* expression in tumours from different geographical locations. Exposure to carcinogens has been shown in animal model carcinogenesis systems to affect levels of *mdr* expression (Gottesman, 1988). Differences in proportions of tumours with detectable *mdr* expression may reflect variations in tumour aetiology and carcinogen exposure in different geographical locations. We have also detected *mdrl* expression in squamous cell carcinomas of the head and neck and in samples from AML patients. All of these samples were from patients who had not received chemotherapy.

Direct evidence for involvement of *mdrl* expression in clinical response of tumours to chemotherapy would require a prospective clinical trial. Drug resistance in multidrug resistant cell lines can be overcome *in vitro* using agents such as verapamil and quinidine which are thought to act by competitively binding to the P-glycoprotein (Safa, 1988). High expression of P-glycoprotein in breast tumours suggest that trials using such reversing agent in conjunction with chemotherapy may be appropriate for breast cancer. For some drugs, such as quinidine, the levels which are clinically achievable are equivalent to those which are effective *in vivo* and a controlled clinical trial in breast cancer using quinidine is underway in our department.

We would like to thank Drs M. Gottesman and I. Pastan (NIH, Bethesda) for making available to us the plasmid MDR5A and Dr M. Muramatsu (University of Tokyo, Tokyo) for the plasmid GPi. We are also very grateful to a number of surgeons and pathologists in Glasgow for helping with the collection of tumour samples, including Prof. D. George, Drs A.-M. McNicol, C. Mcardle, G. Smith, D. Souter, A. MacKay, C. MacKay and I. Brown and also Dr C. Marshall (Chester Beatty Laboratories, London) for the AML samples. We would also like to thank Prof. S.B. Kaye and Dr I. Freshney for very useful discussion. This work was supported by the Cancer Research Campaign.

### References

BALMAIN, A., KRUMLAUF, R., VASS, J.K. & BIRNIE, G.D. (1982). Cloning and characterisation of the abundant cytoplasmic 75 RNA from mouse cells. *Nucl. Acids Res.*, **10**, 425.

BONADONNA, G., MORFARDINI, S., DE LENA, M., FOSSATI-BELLANI, F. & BERETTA, G. (1970). Phase 1 and preliminary phase II evaluation of adriamycin. *Cancer Res.*, **30**, 2572.

BLUM, R.H. & CARTER, S.K. (1974). Adriamycin – a new anticancer drug with significant clinical activity. *Ann. Intern. Med.*, **80**, 249.

CHEN, C.-J., CHIN, J.E., UEDA, K. & 4 others (1986). Internal duplication and homology with bacterial transport proteins in the mdrl (P-glycoprotein) gene from multidrug-resistant human cells. *Cell*, **47**, 381.

COWAN, K.H., BATIST, G., TULPULE, A., SINHA, B.K. & MYERS, C.E. (1986). Similar biochemical changes associated with multidrug resistance in human breast cancer cells and carcinogen induced resistance to xenobiotics in rats. *Proc. Natl Acad. Sci. USA*, **83**, 9328.

DEFFIE, A.M., ALAM, T., SENEVIRATNE, C. & 5 others (1988). Multifactorial resistance to adriamycin. *Cancer Res.*, **48**, 3595.

FOJO, A.T., SHEN, D.W., MICKLEY, L.A., PASTAN, I. & GOTTESMAN, M.M. (1987*a*). Intrinsic drug resistance in human kidney cancer is associated with expression of a human multidrug resistance gene. *J. Clin. Oncol.*, **5**, 1922.

FOJO, A., UEDA, K., SLAMON, D.J., POPLACK, D.G., GOTTESMAN, M.M. & PASTAN, I. (1987*b*). Expression of a multidrug resistance gene in human tumours and tissues. *Proc. Natl Acad. Sci. USA*, **84**, 265.

GERLACH, J.H., BELL, D.R., KARAKOUSIS, C. & 5 others (1987). P-glycoprotein in human sarcoma: evidence for multidrug resistance. *J. Clin. Oncol.*, **5**, 1452.

GERLACH, J.H., ENDICOTT, J.A., JURANKA, P.F. & 4 others (1986). Homology between P-glycoprotein and a bacterial hemolysin transport protein suggests a mode for multidrug resistance. *Nature*, **324**, 485.

GOLDSTEIN, L.J., GALSKI, H., FOJO, A. & 11 others (1989). Expression of a multidrug resistance gene in human cancers. *J. Natl Cancer Inst.*, **81**, 116.

GOTTESMAN, M.M. (1988). Multidrug resistance during chemical carcinogenesis: A mechanism revealed? *J. Natl Cancer Inst.*, **80**, 1351.

GUILD, B.C., MULLIGAN, R.C., GROS, P. & HOUSMAN, D.E. (1988). Retroviral transfer of a murine cDNA for multidrug resistance confers pleiotropic drug resistance to cells without prior drug selection. *Proc. Natl Acad. Sci. USA*, **85**, 1595.

HARLEY, C.B. (1987). Hybridisation of oligo (dT) to RNA on nitrocellulose. *Gene Anal. Tech.*, **4**, 17.

JAKOBY, W.B. (1978). The glutathione S-transferases: a group of multifunctional detoxification proteins. *Adv. Enzymol.*, **46**, 383.

KANO, T., SAKAI, M. & MURAMATSU, M. (1987). Structure and expression of a human class π glutathione S-transferase messenger RNA. *Cancer Res.*, **47**, 5620.

KARTNER, N., EVERNDER-PORELLA, E., BRADLEY, G. & LING, V. (1985). Detection of P-glycoprotein in multidrug-resistant cell lines by monoclonal antibodies. *Nature*, **316**, 820.

KAYE, S.B. (1988). The multidrug resistance phenotype. *Br. J. Cancer*, **58**, 691.

KRIEG, P., ANTMANN, E. & SAUER, G. (1983). The simultaneous extraction of high molecular weight DNA and of RNA from solid tumours. *Ann. Biochem.*, **134**, 288.

MERKEL, D.E., FUQUA, S.A.W., TANDON, A.K., HILL, S.M., BUZDAR, A.V. & MCGUIRE, W.L. (1989). Electrophoretic analysis of 248 clinical breast cancer specimens for P-glycoprotein overexpression or amplification. *J. Clin. Oncol.*, **7**, 1129.

MOSCOW, J.A., FAIRCHILD, C.R., MADDEN, M.J. & 7 others (1989). Expression of anionic glutathione-S-transferase and P-glycoprotein genes in human tissues and tumours. *Cancer Res.*, **49**, 1422.

MOSCOW, J.A., TOWNSEND, A.J., GOLDSMITH, M.E. & 7 others (1988). Isolation of the human anionic glutathione S-transferase cDNA and the relation of its gene expression to estrogen-receiptor content in primary breast cancer. *Proc. Natl Acad. Sci. USA*, **85**, 6518.

O'BRYAN, R.M., BAKER, L.H., GOTTLEIB, J.E. & 6 others (1977). Dose response evaluation of adriamycin in human neoplasia. *Cancer*, **39**, 1940.

O'BRYAN, R.M., LUCE, J.K., TALLEY, R.W., GOTTLIEB, J.A., BAKER, L.H. & BONADONNA, G. (1973). Phase II evaluation of adriamycin in human neoplasia. *Cancer*, **32**, 1.

RIORDAN, J.R., DEUCHARTS, K., KARTNER, N., ALAN, N., TRENT, J. & LING, V. (1985). Amplification of P-glycoprotein genes in multidrug-resistant mammalian cell lines. *Nature*, **316**, 817.

ROGAN, A.M., HAMILTON, T.C., YOUNG, R.C., KLECHER, R.W. & OZOLS, R.F. (1984). Reversal of adriamycin resistance by verapamil in human ovarian cancer. *Science*, **224**, 994.

SAFA, A.R. (1988). Photoaffinity labeling of the multidrug resistance related P-glycoprotein with photoactive analogs of verapamil. *Proc. Natl Acad. Sci. USA*, **85**, 7187.

SALMON, S.E., GORGA, T.M., MILLER, T., SCHEPER, R. & DALTON, W.S. (1989). Prediction of doxorubicin resistance in vitro in myeloma, lymphoma and breast cancer by P-glycoprotein staining. *J. Natl Cancer Inst.*, **81**, 696.

SCOTTO, K.W., BIEDLER, J.L. & MELERA, P.W. (1986). Amplification and expression of genes associated with multidrug resistance in mammalian cells. *Science*, **232**, 751.

SHEN, D.W., FOJO, A., CHIN, J.E. & 4 others (1986). Human multidrug-resistant cell lines: increased *mdr1* expression can precede gene amplification. *Science*, **232**, 643.

SMITH, H.S., HACKETT, A., LAN, S. & STAMPFER, M.R. (1981*a*). Use of an efficient method for culturing human mammary epithelial cells to study adriamycin sensitivity. *Cancer Chemother. Pharmacol.*, **6**, 237.

SMITH, H.S., LAN, S., CERIANI, R., HACKETT, A.J. & STAMPFER, M.R. (1981*b*). Clonal proliferation of cultured nonmalignant and malignant human breast epithelia. *Cancer Res.*, **41**, 4637.

SMITH, H.S., LIPPMAN, M.E., HILLER, A.J., STAMPFER, M.R. & HACKETT, A.J. (1985). Response to doxorubicin of cultured normal and cancerous human mammary epithelial cells. *J. Natl Cancer Inst.*, **74**, 341.

STALLARD, S., MORRISON, J.G., GEORGE, W.D. & KAYE, S.B. (1990). Distribution of doxorubicin to normal breast and tumour tissue in patients undergoing mastectomy. *Cancer Chemother. Pharmacol.*, **25**, 286.

UEDA, K., CASDARELLI, C., GOTTESMAN, M.M. & PASTAN, I. (1987*a*). Expression of a full length cDNA for the human mdr1 gene confers resistance to colchicine, doxorubicin and vinblastine. *Proc. Natl Acad. Sci. USA*, **84**, 3004.

UEDA, K., CLARK, D.P., CHEN, C.-J., RONINSON, I.B., GOTTESMAN, M.M. & PASTAN, I. (1987*b*). The human multidrug resistance (*mdr1*) gene. *J. Biol. Chem.*, **262**, 505.

VAN DER BLIEK, A.M., BAAS, T., VAN DER VELDE-KOERTS, T. & 6 others (1988). Genes amplified and overexpressed in human multidrug-resistant cell lines. *Cancer Res.*, **48**, 5927.


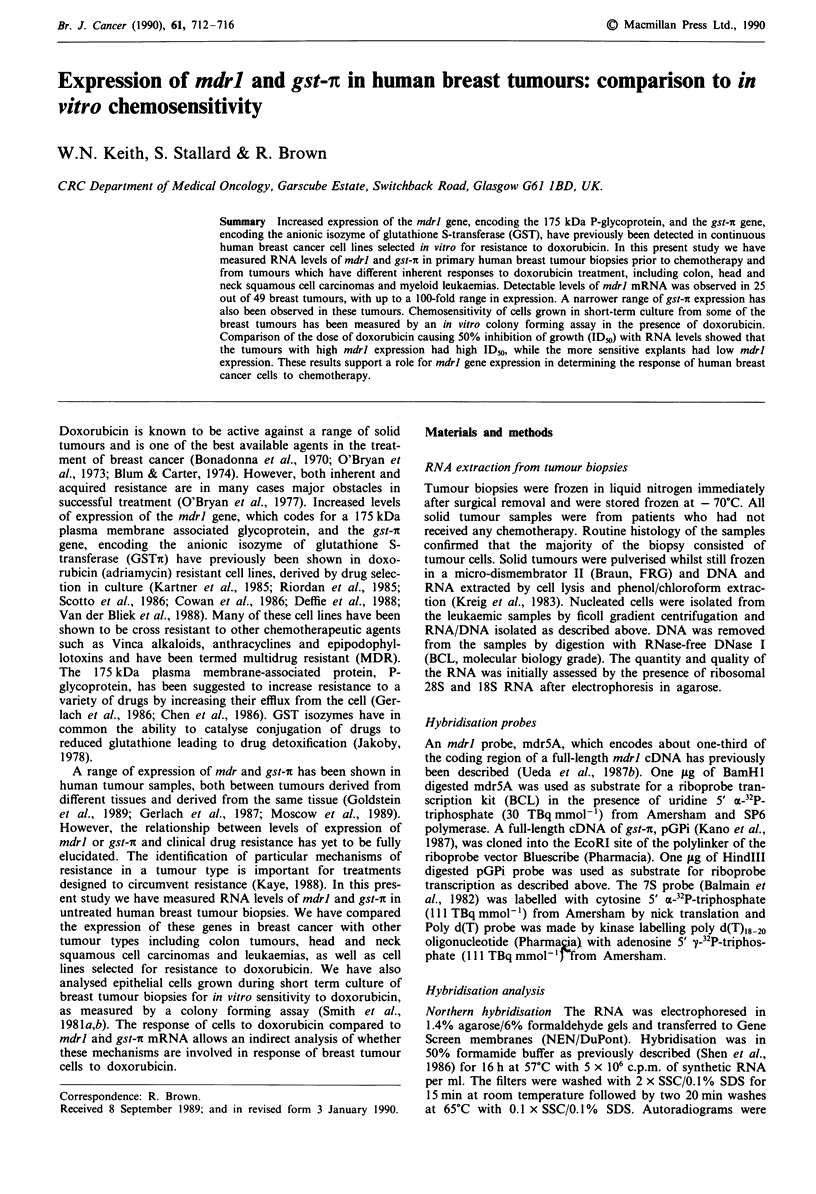

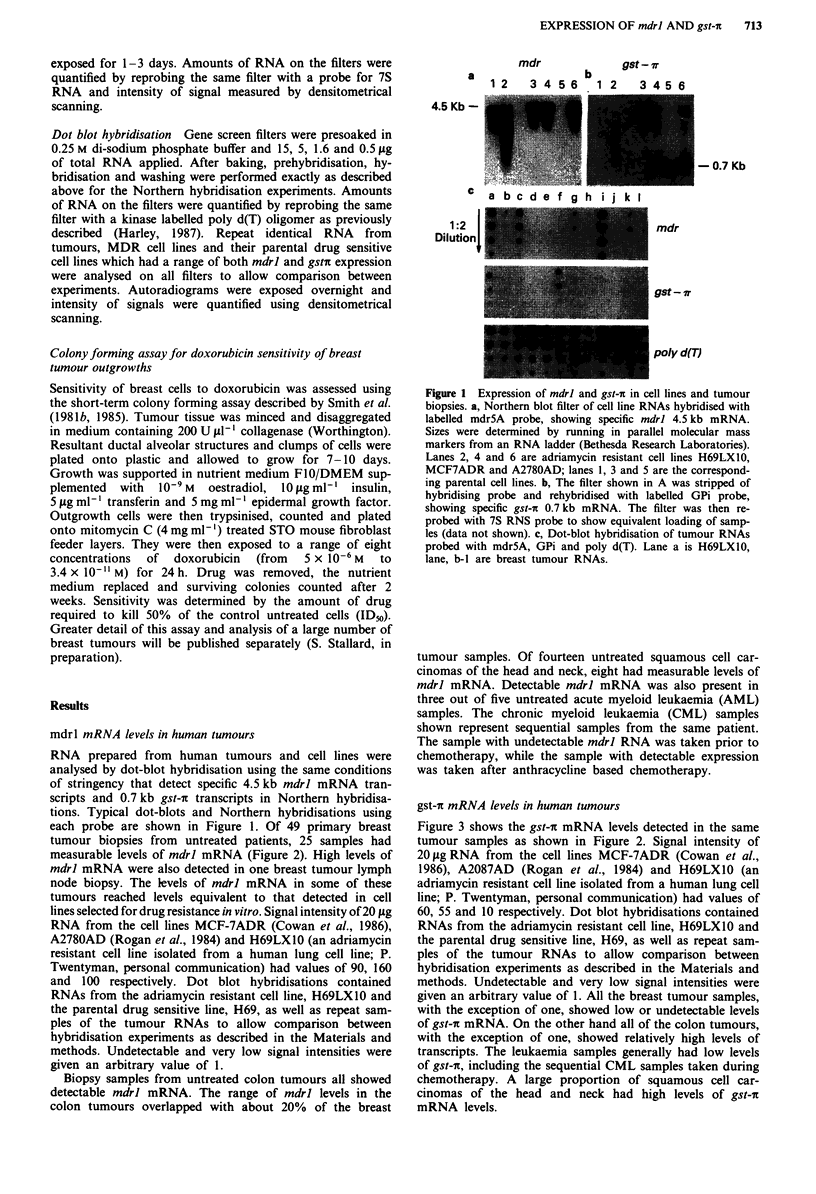

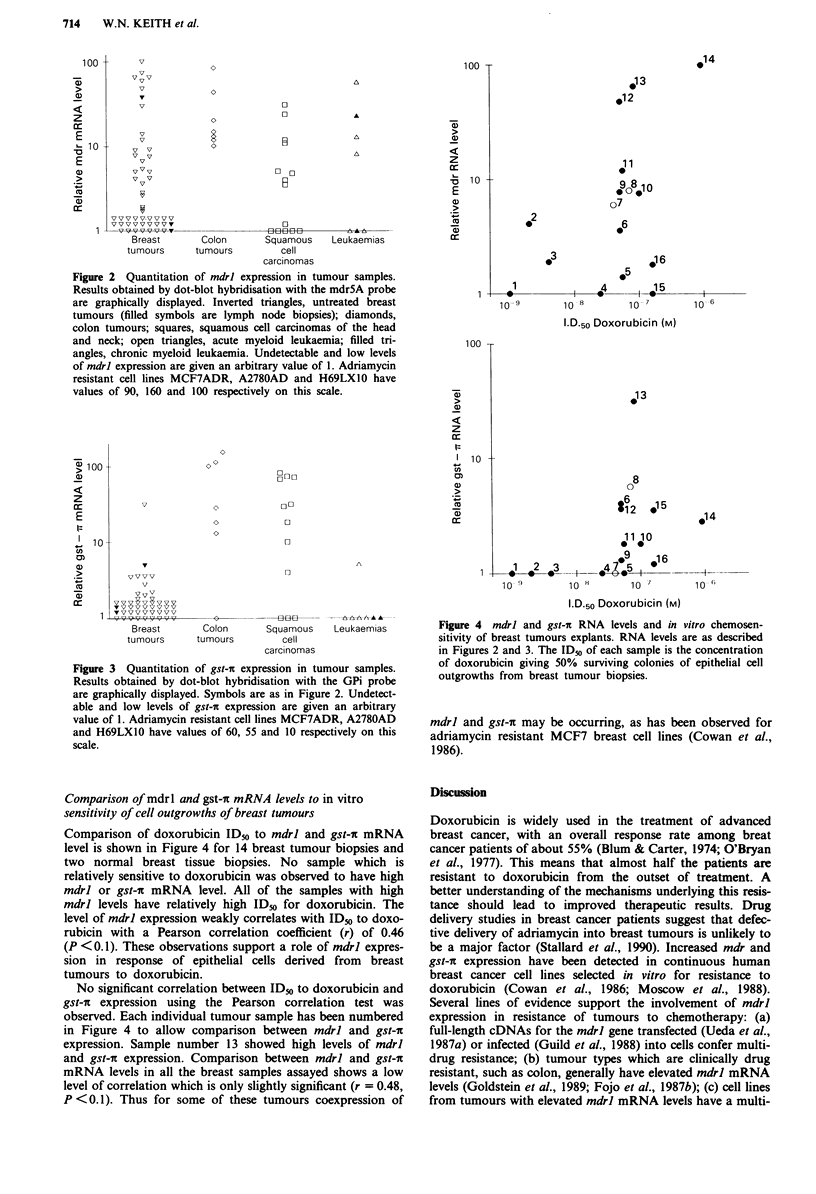

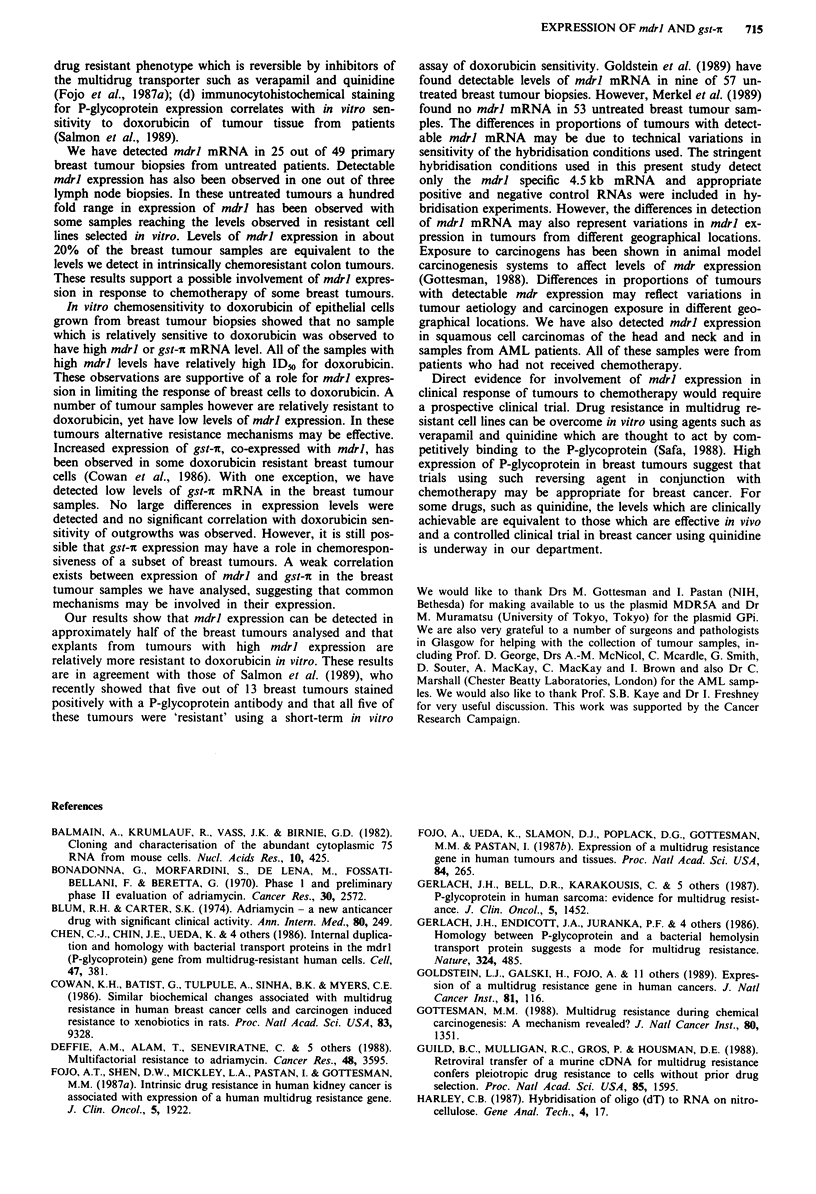

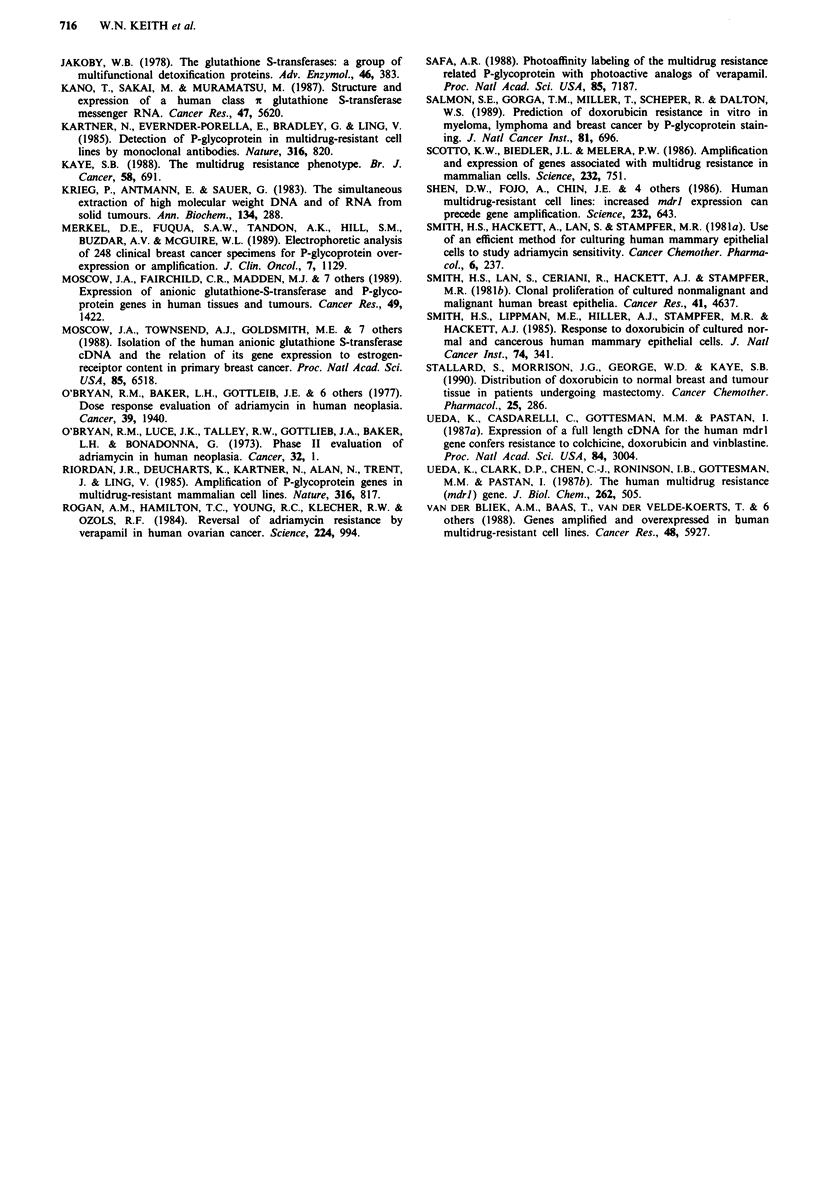


## References

[OCR_00688] Blum R. H., Carter S. K. (1974). Adriamycin. A new anticancer drug with significant clinical activity.. Ann Intern Med.

[OCR_00685] Bonadonna G., Monfardini S., De Lena M., Fossati-Bellani F., Beretta G. (1970). Phase I and preliminary phase II evaluation of adriamycin (NSC 123127).. Cancer Res.

[OCR_00691] Chen C. J., Chin J. E., Ueda K., Clark D. P., Pastan I., Gottesman M. M., Roninson I. B. (1986). Internal duplication and homology with bacterial transport proteins in the mdr1 (P-glycoprotein) gene from multidrug-resistant human cells.. Cell.

[OCR_00697] Cowan K. H., Batist G., Tulpule A., Sinha B. K., Myers C. E. (1986). Similar biochemical changes associated with multidrug resistance in human breast cancer cells and carcinogen-induced resistance to xenobiotics in rats.. Proc Natl Acad Sci U S A.

[OCR_00704] Deffie A. M., Alam T., Seneviratne C., Beenken S. W., Batra J. K., Shea T. C., Henner W. D., Goldenberg G. J. (1988). Multifactorial resistance to adriamycin: relationship of DNA repair, glutathione transferase activity, drug efflux, and P-glycoprotein in cloned cell lines of adriamycin-sensitive and -resistant P388 leukemia.. Cancer Res.

[OCR_00756] Fink D. W., Mirkin B. L. (1987). Effects of chemical sympathectomy in neonatal and adult mice on C-1300 neuroblastoma tumor growth and catecholamine content.. Cancer Res.

[OCR_00708] Fojo A. T., Shen D. W., Mickley L. A., Pastan I., Gottesman M. M. (1987). Intrinsic drug resistance in human kidney cancer is associated with expression of a human multidrug-resistance gene.. J Clin Oncol.

[OCR_00714] Fojo A. T., Ueda K., Slamon D. J., Poplack D. G., Gottesman M. M., Pastan I. (1987). Expression of a multidrug-resistance gene in human tumors and tissues.. Proc Natl Acad Sci U S A.

[OCR_00720] Gerlach J. H., Bell D. R., Karakousis C., Slocum H. K., Kartner N., Rustum Y. M., Ling V., Baker R. M. (1987). P-glycoprotein in human sarcoma: evidence for multidrug resistance.. J Clin Oncol.

[OCR_00725] Gerlach J. H., Endicott J. A., Juranka P. F., Henderson G., Sarangi F., Deuchars K. L., Ling V. (1986). Homology between P-glycoprotein and a bacterial haemolysin transport protein suggests a model for multidrug resistance.. Nature.

[OCR_00731] Goldstein L. J., Galski H., Fojo A., Willingham M., Lai S. L., Gazdar A., Pirker R., Green A., Crist W., Brodeur G. M. (1989). Expression of a multidrug resistance gene in human cancers.. J Natl Cancer Inst.

[OCR_00741] Guild B. C., Mulligan R. C., Gros P., Housman D. E. (1988). Retroviral transfer of a murine cDNA for multidrug resistance confers pleiotropic drug resistance to cells without prior drug selection.. Proc Natl Acad Sci U S A.

[OCR_00747] Harley C. B. (1987). Hybridization of oligo(dT) to RNA on nitrocellulose.. Gene Anal Tech.

[OCR_00753] Jakoby W. B. (1978). The glutathione S-transferases: a group of multifunctional detoxification proteins.. Adv Enzymol Relat Areas Mol Biol.

[OCR_00761] Kartner N., Evernden-Porelle D., Bradley G., Ling V. Detection of P-glycoprotein in multidrug-resistant cell lines by monoclonal antibodies.. Nature.

[OCR_00766] Kaye S. B. (1988). The multidrug resistance phenotype.. Br J Cancer.

[OCR_00770] Krieg P., Amtmann E., Sauer G. (1983). The simultaneous extraction of high-molecular-weight DNA and of RNA from solid tumors.. Anal Biochem.

[OCR_00775] Merkel D. E., Fuqua S. A., Tandon A. K., Hill S. M., Buzdar A. U., McGuire W. L. (1989). Electrophoretic analysis of 248 clinical breast cancer specimens for P-glycoprotein overexpression or gene amplification.. J Clin Oncol.

[OCR_00783] Moscow J. A., Fairchild C. R., Madden M. J., Ransom D. T., Wieand H. S., O'Brien E. E., Poplack D. G., Cossman J., Myers C. E., Cowan K. H. (1989). Expression of anionic glutathione-S-transferase and P-glycoprotein genes in human tissues and tumors.. Cancer Res.

[OCR_00787] Moscow J. A., Townsend A. J., Goldsmith M. E., Whang-Peng J., Vickers P. J., Poisson R., Legault-Poisson S., Myers C. E., Cowan K. H. (1988). Isolation of the human anionic glutathione S-transferase cDNA and the relation of its gene expression to estrogen-receptor content in primary breast cancer.. Proc Natl Acad Sci U S A.

[OCR_00794] O'Bryan R. M., Baker L. H., Gottlieb J. E., Rivkin S. E., Balcerzak S. P., Grumet G. N., Salmon S. E., Moon T. E., Hoogstraten B. (1977). Dose response evaluation of adriamycin in human neoplasia.. Cancer.

[OCR_00799] O'Bryan R. M., Luce J. K., Talley R. W., Gottlieb J. A., Baker L. H., Bonadonna G. (1973). Phase II evaluation of adriamycin in human neoplasia.. Cancer.

[OCR_00804] Riordan J. R., Deuchars K., Kartner N., Alon N., Trent J., Ling V. Amplification of P-glycoprotein genes in multidrug-resistant mammalian cell lines.. Nature.

[OCR_00809] Rogan A. M., Hamilton T. C., Young R. C., Klecker R. W., Ozols R. F. (1984). Reversal of adriamycin resistance by verapamil in human ovarian cancer.. Science.

[OCR_00814] Safa A. R. (1988). Photoaffinity labeling of the multidrug-resistance-related P-glycoprotein with photoactive analogs of verapamil.. Proc Natl Acad Sci U S A.

[OCR_00819] Salmon S. E., Grogan T. M., Miller T., Scheper R., Dalton W. S. (1989). Prediction of doxorubicin resistance in vitro in myeloma, lymphoma, and breast cancer by P-glycoprotein staining.. J Natl Cancer Inst.

[OCR_00825] Scotto K. W., Biedler J. L., Melera P. W. (1986). Amplification and expression of genes associated with multidrug resistance in mammalian cells.. Science.

[OCR_00830] Shen D. W., Fojo A., Chin J. E., Roninson I. B., Richert N., Pastan I., Gottesman M. M. (1986). Human multidrug-resistant cell lines: increased mdr1 expression can precede gene amplification.. Science.

[OCR_00835] Smith H. S., Hackett A. J., Lan S., Stampfer M. R. (1981). Use of an efficient method for culturing human mammary epithelial cells to study adriamycin sensitivity.. Cancer Chemother Pharmacol.

[OCR_00841] Smith H. S., Lan S., Ceriani R., Hackett A. J., Stampfer M. R. (1981). Clonal proliferation of cultured nonmalignant and malignant human breast epithelia.. Cancer Res.

[OCR_00846] Smith H. S., Lippman M. E., Hiller A. J., Stampfer M. R., Hackett A. J. (1985). Response to doxorubicin of cultured normal and cancerous human mammary epithelial cells.. J Natl Cancer Inst.

[OCR_00852] Stallard S., Morrison J. G., George W. D., Kaye S. B. (1990). Distribution of doxorubicin to normal breast and tumour tissue in patients undergoing mastectomy.. Cancer Chemother Pharmacol.

[OCR_00858] Ueda K., Cardarelli C., Gottesman M. M., Pastan I. (1987). Expression of a full-length cDNA for the human "MDR1" gene confers resistance to colchicine, doxorubicin, and vinblastine.. Proc Natl Acad Sci U S A.

[OCR_00864] Ueda K., Clark D. P., Chen C. J., Roninson I. B., Gottesman M. M., Pastan I. (1987). The human multidrug resistance (mdr1) gene. cDNA cloning and transcription initiation.. J Biol Chem.

[OCR_00869] Van der Bliek A. M., Baas F., Van der Velde-Koerts T., Biedler J. L., Meyers M. B., Ozols R. F., Hamilton T. C., Joenje H., Borst P. (1988). Genes amplified and overexpressed in human multidrug-resistant cell lines.. Cancer Res.

